# A Comprehensive Systematic Review and Meta-Analysis to Unravel the Noise-Dementia Nexus

**DOI:** 10.3389/phrs.2025.1607355

**Published:** 2025-06-16

**Authors:** Soonmyung A. Hwang, Aditi Singhvi, Lokesh Patil, Kimiya Gohari, Maayan Yitshak Sade, Elena Colicino, Melissa D. Aldridge, Andrea A. Baccarelli, Itai Kloog, Joel Schwartz, R. Sean Morrison, Robert O. Wright, Henrik Bøggild, Ali Sheidaei, Pablo Knobel

**Affiliations:** ^1^ Icahn School of Medicine at Mount Sinai, New York, NY, United States; ^2^ Department of Environmental Medicine, Icahn School of Medicine at Mount Sinai, New York, NY, United States; ^3^ Department of Obstetrics, Gynecology and Reproductive Medicine, Renaissance School of Medicine at Stony Brook University, Stony Brook, NY, United States; ^4^ Brookdale Department of Geriatrics and Palliative Medicine, Icahn School of Medicine at Mount Sinai, New York, NY, United States; ^5^ Office of the Dean, Harvard T.H. Chan School of Public Health, Boston, MA, United States; ^6^ Institute for Exposomic Research, Icahn School of Medicine at Mount Sinai, New York, NY, United States; ^7^ Department of Environmental Health, Harvard T.H. Chan School of Public Health, Boston, MA, United States; ^8^ James J. Peters Veterans Affairs Medical Center, Bronx, NY, United States; ^9^ Department of Health Science and Technology, Aalborg University, Aalborg, Denmark; ^10^ Department of Epidemiology and Biostatistics, Tehran University of Medical Sciences, Tehran, Iran

**Keywords:** Alzheimer’s disease and related dementias (ADRD), air pollution, exposome, noise, meta-analysis

## Abstract

**Objectives:**

As the aging population grows, Alzheimer’s disease and related dementias (ADRD) present a major public health challenge. Environmental noise, linked to stress and sleep disruption, may increase ADRD risk. We aimed to summarize the research literature on long-term noise exposure and ADRD.

**Methods:**

We conducted a systematic review and meta-analysis of studies investigating the association of long-term (≥1 year) noise exposure and ADRD assessed with standardized diagnostic criteria. Two reviewers independently screened studies, extracted data, and assessed risk of bias. Eligible studies reported hazard ratios (HR) or similar effect estimates with confidence intervals.

**Results:**

A multilevel random-effects meta-analysis of six longitudinal studies using 13 effect sizes found a significant association between long-term noise exposure and incident ADRD (HR: 1.15, 95% CI: 1.03–1.28). Interaction effects between noise source and dementia subtype were not statistically significant.

**Conclusion:**

Long-term noise exposure may contribute to ADRD risk. Heterogeneity between studies highlights the need for standardized exposure assessment and consideration of other environmental factors. Future research should include the exposome approach for identifying environmental drivers of dementia.

## Introduction

Dementia refers to a collection of diseases characterized by a continuous and irreversible deterioration of cognitive abilities, frequently linked to aging. Alzheimer’s disease (AD) accounts for 60%–80% of all dementia cases, with the rest being attributed to various other forms of dementia [1]. Around 5%–10% of dementia cases are due to cerebrovascular or vascular dementia (VaD), 3%–10% to frontotemporal degeneration (FTD), 3%–13% to hippocampal sclerosis (HS), 5% to Lewy body disease (LBD), and 4% to Parkinson’s disease (PD) [2]. Interestingly, about half of all dementia cases are linked to multiple causes and are thus classified as mixed dementia. Alzheimer’s disease and related dementias (ADRD) include a variety of neurodegenerative disorders that impact cognitive abilities like memory, language, reasoning, and behavior. These disorders are categorized based on their root causes, symptoms, and the patterns of brain damage they cause. In 2019, ADRD had an economic impact of 55 million USD, with projections estimating this figure to rise to 82 million USD by 2030 and 152 million USD by 2050 [3, 4]. In 2015, the global cost of dementia was estimated to be around 818 billion USD, representing 1.1% of the world’s GDP. This amount is expected to rise to 2 trillion USD by 2030 [5]. However, these cost projections do not entirely reflect the strain on families.

Noise refers to an unwanted and/or harmful sound that disrupts regular human activities, including communication, sleep, work, or leisure [6]. Noise can be categorized into various subtypes depending on its origin, features, and impacts. Some prevalent subtypes of noise include environmental noise, occupational noise, transportation noise, and recreational noise [7]. Extended exposure to any form of noise can significantly affect human health and wellbeing, leading to issues like hearing impairment, irritation, stress, heart diseases, cognitive dysfunction, and sleep disruptions [8]. A 2011 report by the World Health Organization on the European Union and Western European countries estimated that the disability-adjusted life-years (DALY) lost due to environmental noise were as follows: 61,000 years for ischemic heart disease, 45,000 years for cognitive impairment in children, 903,000 years for sleep disturbances, 22,000 years for tinnitus, and 587,000 years for annoyance [9]. In recent years, cognitive impairment and dementia have emerged as potential effects of noise exposure that are garnering increased focus [10]. Accumulating research indicates that noise could potentially contribute to ADRD, either directly or indirectly. The suggested biological pathways through which noise impacts health involve the stimulation of the autonomic nervous system and the endocrine system, resulting from stress reactions induced by noise [10, 11]. Nighttime noise can cause sleep disruptions and fragmented sleep, which have additional connections to endothelial dysfunction, heightened oxidative stress, changes in the immune system, and escalated systemic inflammation [12–15]. These factors are considered to be early indicators in the development of dementia and AD [16, 17]. Nonetheless, the evidence remains uncertain, and the fundamental processes are not completely comprehended.

To our knowledge, only one systematic review and meta-analysis on the relationship between dementia and noise exposure exists, conducted by Meng et al. [18]. The aforementioned study considered publications up until 18 September 2021. However, it is important to note that the study also included data on mild cognitive impairment (MCI) in its meta-analysis, an outcome that differs significantly from clinical dementia. Additionally, the study factored in occupational noise exposure, which complicates the interpretation and generalizability of the overall results in terms of environmental noise exposure and its association with dementia.

The objective of this systematic review and meta-analysis is to identify and assess existing research regarding the association between long-term noise exposure and ADRD, including its subtypes. Additionally, we calculated the combined effect size of noise exposure on the risk of ADRD and its subtypes, taking into account diverse study designs, populations, and exposure measurements.

## Methods

The systematic review and meta-analysis followed Preferred Reporting Items for Systematic Reviews and Meta-Analyses (PRISMA) guidelines [19]. It has been registered in the International Register of Systematic Review Protocols (PROSPERO) (CRD42023463914).

### Search Strategy

Electronic databases (PubMed, SCOPUS, Web of Science) were systematically searched without restriction in year of publication using the following search strategy on August 1, 2023. Relevant keyword search terms with a combination of Boolean operators (AND, OR, NOT) were utilized in our searches (Supplementary Table S1).

### Eligibility Criteria

#### Inclusion Criteria

We included original research published in peer-reviewed journals that involved adult (18+) human populations, were published in the English language, and had full text available. Our selection process followed a two-level approach. Initially, we sought to include only longitudinal cohort and case-control designs to focus on studies that could establish temporality between noise exposure and ADRD outcomes. However, given the limited number of eligible studies in this emerging field, we subsequently broadened our inclusion criteria to also consider cross-sectional studies. Only studies that provided original data on the relationship between noise and ADRD in the form of odds ratios, relative risks, or hazard ratios, along with their corresponding confidence intervals, were considered. Studies that evaluated noise exposure at the residential level for a minimum duration of 1 year to account for long-term effects and assessed ADRD outcomes using standardized diagnostic criteria or validated tools were included.

#### Exclusion Criteria

We excluded studies that did not clearly state the diagnostic criteria or instruments used to assess ADRD outcomes. This is crucial to ensure the validity and reliability of the results. Studies that used non-standardized or non-validated methods to assess ADRD outcomes were also not considered, as these methods may not accurately reflect the true prevalence or incidence of ADRD in the studied populations.

### Study Selection

We utilized Covidence to facilitate the process of screening and extracting data from our search results [20]. Following the inclusion and exclusion criteria of our search strategy, each record was reviewed by at least two individuals among three independent reviewers (SH, AS, and LP) independently evaluated the titles and abstracts of the retrieved literature to determine their suitability. Any disagreements between the reviewers were settled by a third reviewer (HA). Publications that passed the initial screening based on their eligibility criteria were then subjected to a comprehensive review of the full text by at least two reviewers (SH, AS, LP) to verify their eligibility.

### Data Extraction

The following relevant data were then extracted from all eligible studies: study characteristics (publication year, study setting, study design, sample size), demographic characteristics (mean/median age, sex), study exposures, study outcomes, and confounding variables. Further, reference lists of included studies and review articles were examined for potential articles that may have not been captured by our search strategy.

### Risk of Bias Assessment

Each included article underwent a risk of bias assessment using the Newcastle-Ottawa Scale, a widely used tool for assessing the risk of bias in non-randomized studies, particularly case-control and cohort studies [21]. The NOS employs a “star system” to evaluate a study from three general viewpoints: 1) the selection process for the study groups; 2) the comparability of these groups; and 3) the determination of either the exposure or outcome of interest for case-control or cohort studies, respectively. Regarding the risk of bias, studies that achieve a total score of 7-9 are deemed to have a low risk of bias; a score of 6 points suggests an intermediate risk of bias; a score of 5 points or lower indicates a high risk of bias [21]. At least two independent reviewers out of three (SH, AS, LP) conducted the risk of bias assessment, with a third reviewer (HA) resolving any disagreement that resulted.

### Statistical Analysis

We conducted fixed-effects and random-effects meta-analyses to calculate the overall hazard ratios (HR) with 95% confidence intervals (CI), using the fixed-effects model to assume a common effect size and the random-effects model to account for between-study variability. We evaluated the heterogeneity among the results of the studies using the I^2^ statistic, and when substantial (I^2^ >50%), we prioritized the results from the random-effects model for more conservative and generalizable estimates.

To ensure consistency across studies, HRs were standardized to represent an increment of 10 decibels (dB) in noise exposure. For studies that reported categorical HRs, we estimated a linear hazard ratio per 10 dB increase by assigning representative values to each noise category based on their medians. A linear model was then fitted to the reported HRs against these median exposure levels, and the slope was extracted as the log-HR per 1 dB increase. This value was multiplied by 10 to obtain the HR per 10 dB increase, facilitating comparability across studies.

A multilevel random-effects meta-analysis was conducted to account for the non-independence of multiple effect sizes arising from the same study and disease subtype. The model incorporated two levels of random effects: between-study heterogeneity and within-study clustering across outcome types. This hierarchical structure allowed for appropriate modeling of within- and between-study variance components.

Two meta-regression models were specified to explore potential sources of heterogeneity. The first included an interaction term between noise exposure source (road traffic, railway, or environmental/residential) and dementia outcome type (all-cause dementia, Alzheimer’s disease, or vascular dementia). The second, an additive model, included both variables as main effects. Model fit was evaluated using the Akaike Information Criterion (AIC), and the significance of moderators was tested using omnibus Wald-type Q statistics. Robustness of the pooled estimates was evaluated via a leave-one-out sensitivity analysis, in which the meta-analysis was re-estimated iteratively after omitting each study. The resulting changes in pooled and confidence intervals were compared across models to identify potential influential studies. Potential small-study effects and publication bias were assessed visually using funnel plots and statistically using a regression-based test for funnel plot asymmetry. These assessments were repeated within subgroups defined by noise exposure type and dementia subtype. Due to the absence of significant asymmetry, no further adjustment procedures were applied.

All statistical analyses were performed in R (v4.4.2).

## Results

**FIGURE 1:**
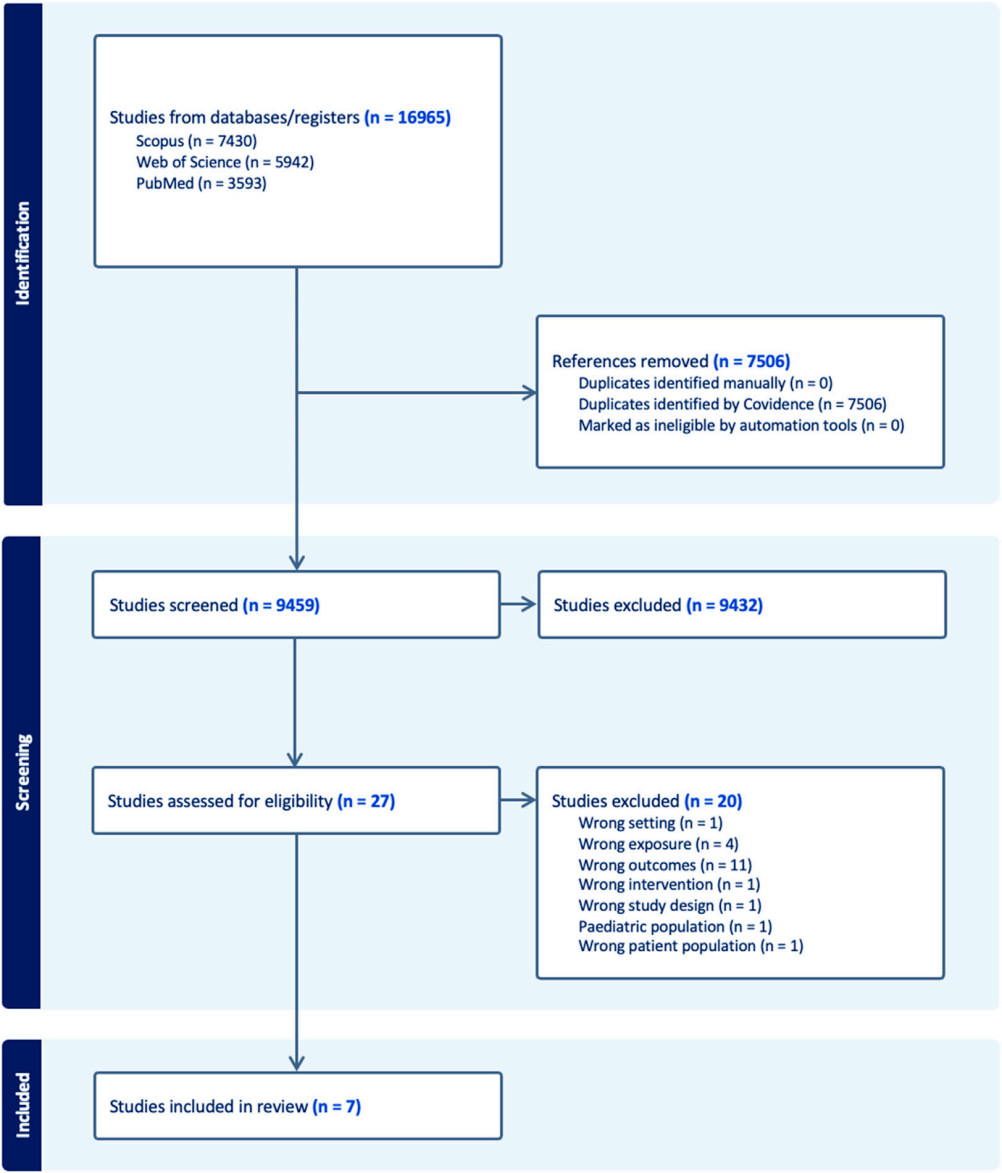
Preferred Reporting Items for Systematic Reviews and Meta-Analyses (PRISMA) chart outlining screening process (New York, United States. 2025).

Our initial search yielded a total of 16,965 records. We then proceeded to remove duplicates from this pool, which amounted to 7,506 records. This left us with 9,459 unique records to evaluate. The next step involved screening these records based on their titles and abstracts. This process helped us identify potentially relevant studies for our review. At the end of this screening phase, we found 27 records that appeared to meet our eligibility criteria. These 27 records underwent a more detailed evaluation, where we reviewed the full text of each study. During this full-text review, we excluded 20 of these records. Of these excluded records, 11 did not have our outcomes of interest, five studies did not have noise as exposure, two studies had undesired patient populations, one study had an ecological study design, and one study had both an undesired outcome and did not have noise as exposure. Further details outlining the specific reasons for these exclusions can be referenced in Supplementary Table S2. After this predetermined process, we were left with seven records that met all our criteria and were deemed suitable for inclusion in our systematic review (Figure 1). Of these seven studies, six studies were the basis of our meta-analysis and their relevant details are provided in Table 1.

**TABLE 1:** List of included studies (New York, United States, 2025).

Author	Year	Country	Study design	Noise type	Noise exposure assessment method	Type of Dementia(s)	Adjusted covariates	NOS
Cantuaria et al.	2021	Denmark	PCO	Road, railway	Nordic prediction method, Nord2000 model	Dementia, AD, VaD, Parkinson disease dementia	Civil status, country of origin, income, occupational status, highest attained education, population density, neighborhood level SES, building type, high quality green space, PM_2.5_, nitrogen dioxide	8
Carey et al.	2018	U.K.	RCO	Road	TRAffic Noise EXposure (TRANEX)	Dementia, AD, VaD	Age, sex, ethnicity, smoking, BMI, ischemic heart disease, stroke, heart failure, diabetes, IMD decile, NO_2_, PM_2.5_	8
Andersson et al.	2018	Sweden	PCO	Environmental/residential	Umeå Municipality Noise Survey	Dementia	Baseline age, education, physical activity, smoking, sex, body mass index, waist-hip ratio, alcohol use, ApoE4, diabetes, hypertension, stroke	8
Weuve et al.	2021	U.S.A.	PCO	Environmental/residential	Universal kriging model developed for the Chicago area	AD	Calendar time, baseline age, age at exam, sex, race/ethnicity, income, education, neighborhood SES, smoking, alcohol use, NOx, ApoE4	8
Yuchi et al.	2020	Canada	CC	Environmental/residential	CadnaA (Computer Aided Noise Abatement)	AD, non-AD dementia	Comorbidities, household income, education, ethnicity, age, sex	8
Yu et al.	2023	U.S.A.	PCO	Road	Federal Highway Administration Traffic Noise Model	Dementia	Age, sex, education, longest held occupation, neighborhood SES, living county, outdoor physical activity, smoking status, household income at baseline	8
Cole-Hunter et al.	2022	Denmark	PCO	Environmental/residential	NORD2000	Dementia	Smoking, alcohol consumption, working status, marital status, urbanization, municipality-level average income, PM_2.5_, NO_2_	7

Abbreviations: PCO, prospective cohort; RCO, retrospective cohort; CC, case-control; AD, Alzheimer’s disease; VaD, vascular dementia; NOS, Newcastle-Ottawa Scale; SES, socioeconomic status; PM_2.5_, particulate matter 2.5; IMD, Indices of Multiple Deprivation; NO, nitrogen oxides.

### Study Characteristics

The seven studies that were included in our systematic review originated from various countries: two from the United States [22, 23], two from Denmark [24, 25], and one each from Canada [26], Sweden [27], and the United Kingdom [28]. The study designs of these studies varied, with five being prospective cohort studies [22–25, 27], one being a retrospective cohort study [28], and one being a case-control study [26]. We meta-analyzed results from six studies that reported incidence [22, 23, 25–28], excluding a study that reported estimates for mortality due to dementia as an outcome [24, 26]. The dementia subtypes that were examined in these studies included AD, vascular dementia, Parkinson’s disease dementia, and non-Alzheimer’s dementia. As for the sources of noise exposure, the studies considered noise from road/traffic, railway, and residential/environmental sources.

### Exposure Assessment

The reviewed studies utilized diverse methodologies for assessing residential noise exposure, which can be broadly categorized by modeling approach. Deterministic noise propagation models, which rely on physical principles to simulate sound propagation, were used in several studies. The Nordic prediction models and Nord2000, employed in Danish studies by Cantuaria et al. [25] and Cole-Hunter et al. [24], leveraged high-resolution spatial data (1 × 1 m grids), detailed traffic composition (annual average daily traffic, vehicle types, speeds, road classification), railway data (train length, speed, type), and meteorological information (wind, temperature), and accounted for built environment factors (floor height, façade exposure (min/max), noise barriers/beams) to calculate weighted 24-h averages (Lden) with penalties for evening and nighttime noise. These models also incorporated acoustic physics (ground absorption, sound reflections), achieving high precision (±3 dB) in validation. CadnaA, employed in a Canadian study by Yuchi et al. [26], also falls into this category, incorporating road, aircraft, and railway sources and modeling the influence of topography and building reflections. The FHWA Traffic Noise Model, used in a U.S. study by Yu et al. [23], is a simpler deterministic model that focuses on road traffic using vehicle speeds and road attributes but excludes other noise sources and meteorological considerations. Geostatistical modeling, specifically universal kriging, was applied in another U.S. study by Weuve et al. [22], using geographic covariates and participant relocation history to predict noise levels but it relied on daytime noise samples only. Finally, the Swedish study by Andersson et al. [27] used Umeå municipal noise survey model with topography, noise sources, buildings and bodies of water considered modeled on a grid at spatial resolution of 5–10 m in urban areas. While model precision was ±3 dB(A) for noise levels ≥35 dB(A), it did not account for building floor levels and extending grid size to 10–25 m in more quiet rural areas, potentially introducing exposure misclassification.

Despite the varying levels of complexity, several limitations were common across the reviewed studies. Most models relied on static traffic data or traffic volume with linear interpolation, introducing temporal constraints and failing to capture short-term noise variations. Exposure from secondary noise sources like construction sites were also often omitted and individual-level factors, such as bedroom placement, sound insulation, time-activity patterns, and personal mobility, were generally not accounted for, leading to potential exposure misclassification.

### Outcome Assessment

Diagnosing dementia is a complicated and non-standardized procedure, with potential differences in patients’ socioeconomic status, and regional differences in diagnosis rates and services. Misclassification is a limitation especially for the subtypes of dementia [29]. Of the six studies included in our meta-analysis, all looked at incident cases [23, 25, 27, 28]. Cantuaria et. al. defined all-cause dementia as primary or secondary diagnoses of dementia for inpatient and outpatient contacts recorded in the Danish National Patient Register or the Danish Psychiatric Central Register using International Classification of Diseases, Revision 8 (ICD-8) and International Classification of Diseases, Revision 10 (ICD-10) codes along with at least one prescription of anti-dementia drug (donepezil, rivastigmine, galantamine, or memantine) registered in the Danish National Prescription Registry. The authors provided the following information on specific codes in the Supplementary Material: for AD, ICD-8 (290.10), ICD-10 (F00.0, F00.1, F00.2, F00.9, G30.0, G30.1, G30.8, G30.9); for vascular dementia, ICD-8 (293.09, 293.19), ICD-10 (F01.0, F01.1, F01.2, F01.3, F01.8, F01.9); for Parkinson’s disease dementia, ICD-10 (F02.3, G31.8E); for unspecified or other types of dementia, ICD-8 (290.09, 290.11, 290.18, 290.19, 094.19, 292.09), ICD-10 (F03.9, F02.8, F02.0, G31.0B) [25]. Carey et.al. assessed incidence as date of first dementia diagnosis from read codes in primary records in the Clinical Practice Research Datalink database [28]. Information on specific codes used was not provided by the authors in the paper or Supplementary Material. The authors also state using ICD-10 codes in death records for identifying cases where the primary cause of death was dementia. It remains unclear if the authors used information on mortality due to dementia in their analyses or further assessed incidence of dementia in those records. Andersson et. al. used records from 1995 to 2010 of the Betula project, Sweden where dementia was ascertained through a three-phase procedure [27]. Comprehensive analyses of neuropsychological test results, structured interviews, and observations were conducted at baseline and participants suspected of dementia underwent examination by specialists. Criteria for suspicion included MMSE scores ≤23, declining cognitive performance, subjective memory impairment, or deviant observations. Medical records were reviewed, and diagnoses followed DSM-IV criteria, with AD diagnosed according to National Institute of Neurological and Communicative Disorders and Stroke–Alzheimer’s Disease and Related Disorders Association (NINCDS-ADRDA) criteria. Vascular complications were considered for vascular dementia diagnosis. Final diagnoses were made by geriatric specialists. In 2011, they validated their diagnostic method by blinded reevaluation of medical records from participants with dementia diagnosis, finding only 0.4% improperly classified. Yu et al. used data from the Sacramento Area Latino Study on Aging (SALSA) cohort [23]. Participants were screened using Modified Mini–Mental State Examination (3MSE) and Spanish English Verbal Learning Test (SEVLT) for values below 20th percentile or a decline of eight points or more from baseline to be reviewed by team of neurologists. California Alzheimer’s Disease Diagnostic and Treatment Centers (ADDTC) criteria were used to diagnose ischemic vascular dementia and National Institute of Neurological Disorders and Stroke/Alzheimer’s Disease and Related Disorders Association criteria were used to diagnose AD and further confirmed by imaging examination (i.e., MRI) [23, 30].

### Overall Meta-Analysis

**FIGURE 2:**
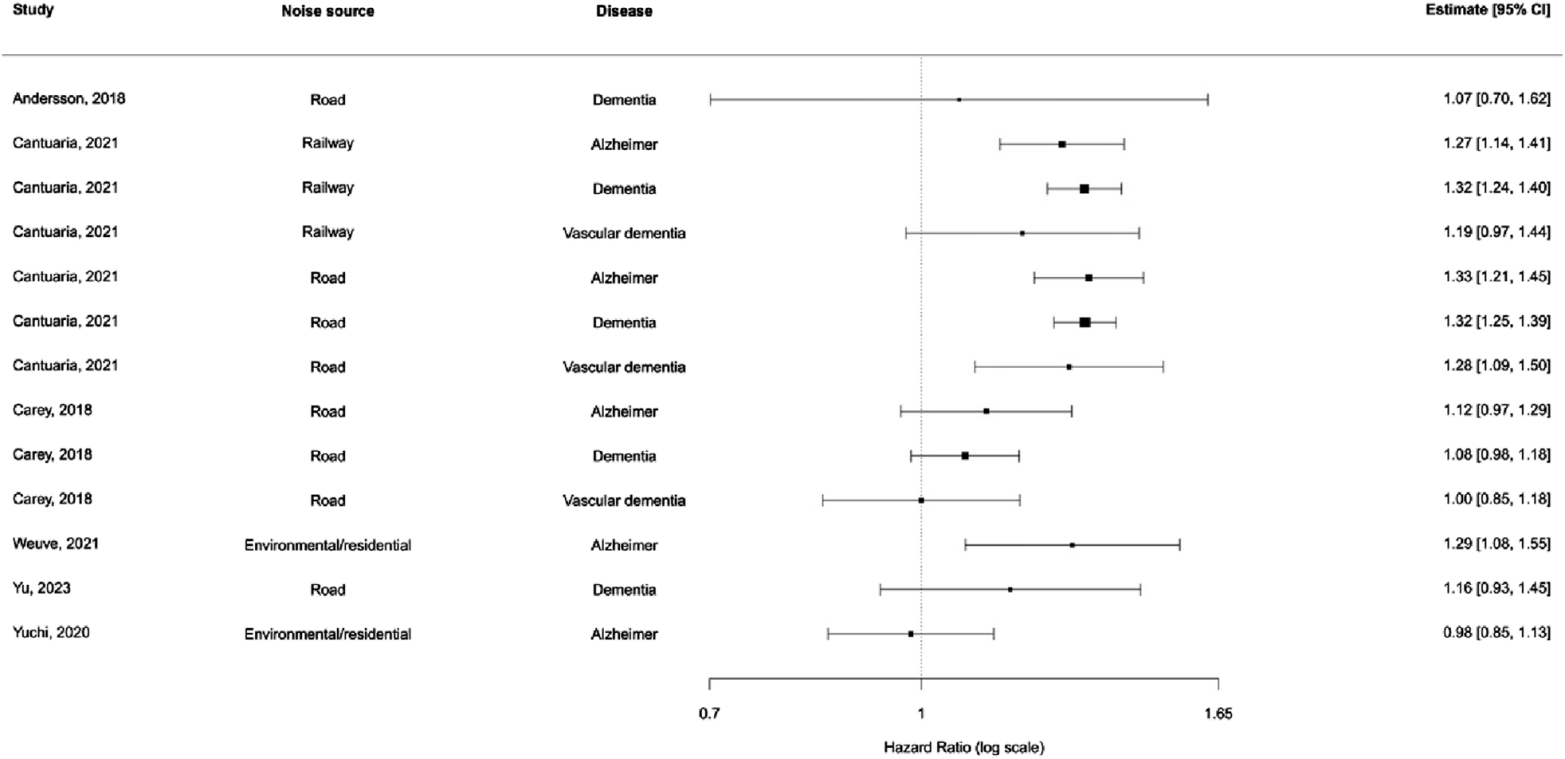
Distribution of effect estimates stratified by noise source and disease subtype (New York, United States. 2025).

We included 13 effect sizes from six unique longitudinal studies assessing the relationship between noise exposure and dementia outcomes (Figure 2). In the baseline multilevel meta-analysis model (random effects by study and outcome), the pooled hazard ratio (HR) was 1.15 (95% CI 1.03–1.28). Adding moderators for noise source and disease type significantly improved model interpretability. However, the interaction between noise source and disease did not meaningfully improve model fit (QM = 2.38, df = 6, p = 0.88), and none of the interaction terms were statistically significant. Therefore, an additive model was selected as the final model for interpretability and parsimony (Supplementary Table S3).

In the additive model, noise from railways and roads showed slightly elevated risks compared to residential noise, though confidence intervals crossed unity. Among disease subtypes, vascular dementia showed the highest pooled HR, but again, no subgroup reached statistical significance (Supplementary Table S3).

**FIGURE 3:**
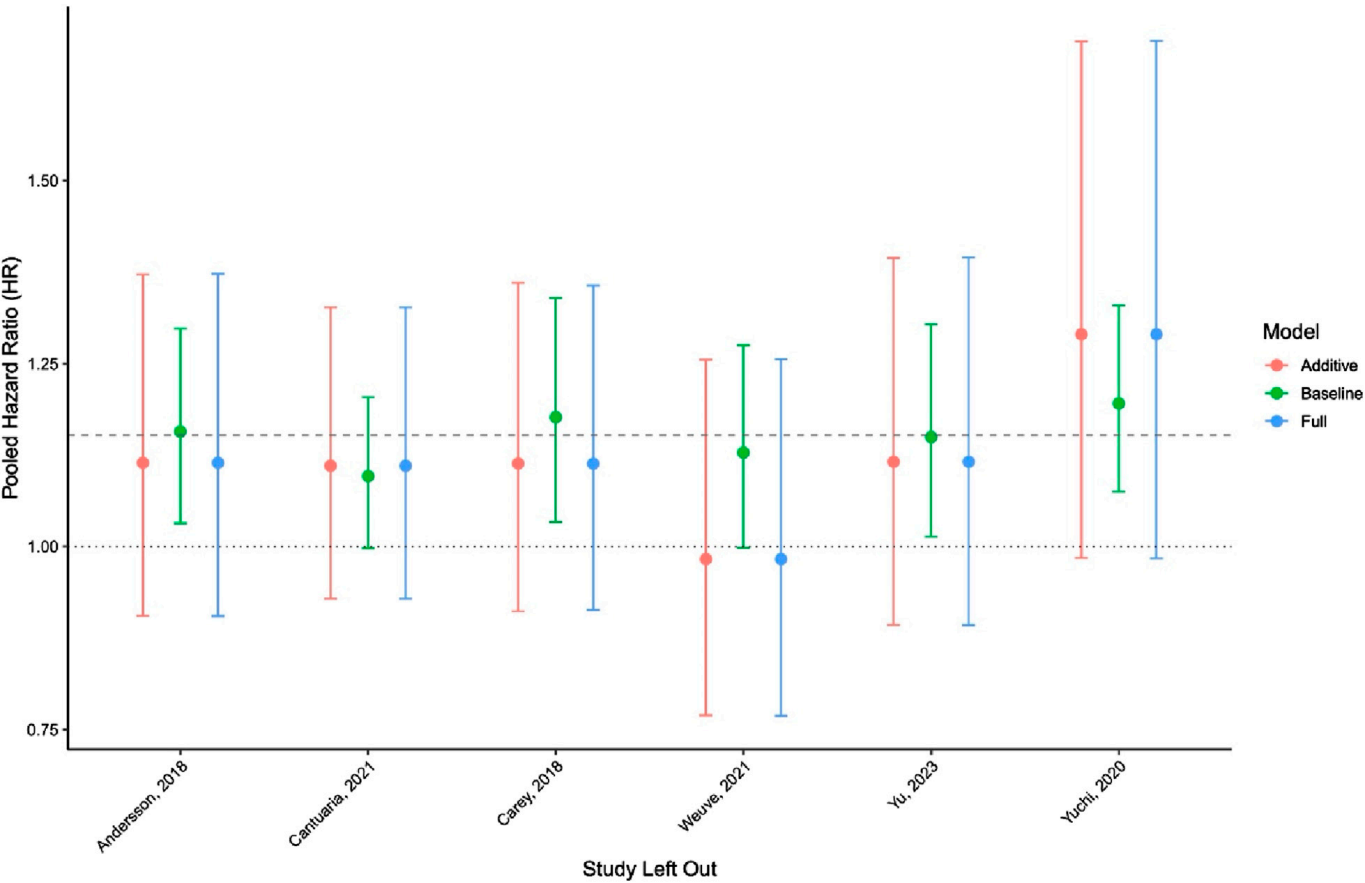
Leave-one-out sensitivity analysis across meta-regression models (New York, United States. 2025).

Sensitivity analyses showed that the pooled HR was robust to exclude any single study. The most significant shifts occurred when omitting Yuchi et al. [26] and Weuve et al. [22], with HR estimates ranging from 0.98 to 1.29 across models (Figure 3). Complete results of the leave-one-out analysis are provided in Supplementary Table S4.

Visual inspection of funnel plots and Egger’s test results showed no evidence of small-study effects or publication bias, either in the main model (Egger’s p = 0.132) or in subgroup analyses by noise source and disease type (all p > 0.24; Supplementary Figure S1). Trim-and-fill analysis was not performed due to a lack of asymmetry.

### Risk of Bias Assessment

All seven studies included in the review were deemed to be of high quality and low risk of bias. Specifically, three studies received the maximum score of 9, reflecting strong performance across all NOS domains. The remaining four studies received a score of 8, with most losing a point in the comparability domain due to partial or unclear adjustment for important covariates such as socioeconomic status and air pollution. Detailed scoring for each domain per study is outlined in Supplementary Table S5 and Supplementary Table S6.

## Discussion

In this systematic review and meta-analysis, we identified and synthesized evidence from seven studies investigating the association between environmental noise exposure and dementia, with six of these studies contributing to our meta-analysis. Across 13 effect sizes from six studies, our findings revealed that higher noise exposure was associated with a modest but statistically significant increase in the risk of ADRD. The pooled hazard ratio in the baseline model indicated a 15% increase in risk (HR = 1.15, 95% CI 1.03–1.28) per 10 dB increase in environmental noise exposure, with consistent patterns observed across model specifications.

Our findings align with and build upon prior reviews that have explored environmental risk factors for dementia. For instance, Meng et al. reported an elevated risk of cognitive impairment per 10 dB increase in noise exposure but included both occupational settings and mild cognitive impairment (MCI), limiting specificity to clinically diagnosed dementia [18]. Jones et al. conducted a broad umbrella review of environmental risk factors for dementia and mild cognitive impairment, that found nine environmental risk factors, such as air pollutants and shift work, associated with higher risk of all-cause dementia [31]. In contrast, our meta-analysis focuses exclusively on long-term residential noise exposure and validated diagnoses of dementia subtypes, allowing for greater precision. This narrower scope enhances the clinical relevance of our findings and supports the growing evidence base linking environmental noise to dementia risk.

The theory supporting this association proposes that prolonged exposure to high noise levels could initiate inflammatory responses, disturb sleep cycles, and cause neurodegenerative changes–mechanisms that are increasing recognized as contributing to the pathogenesis of ADRD subtypes, such as Alzheimer’s disease and vascular dementia [12, 32]. These modifications could conceivably contribute to the development of ADRD [33]. In a meta-analysis of prospective cohort studies, researchers found that individuals with hearing impairment have a significantly increased risk of developing AD. The overall combined relative risk was approximately 4.87 (95% CI 0.90–26.35, p = 0.06) when compared to the control group [34]. It is well recognized that noise exposure is a significant risk factor for hearing impairment and loss [35, 36]. The relationship between noise exposure, hearing loss, and ADRD may involve mediation, where noise leads to hearing impairment, which then increases ADRD susceptibility. Alternatively, hearing loss may modify the effects of noise exposure, either reducing susceptibility due to auditory degradation or amplifying cognitive strain. Clarifying these pathways is essential for understanding how noise exposure and hearing impairment jointly influence ADRD risk [37–39]. It is crucial to acknowledge that the connection between noise exposure and ADRD is intricate and remains to be fully deciphered.

The exposome refers to the comprehensive assessment of all environmental exposures over the human lifespan and their cumulative effects on health [40]. In the context of dementia research, this approach is particularly valuable in disentangling the complex interplay between noise exposure and other co-occurring environmental factors such as air pollution, heat, and proximity to roadways. These exposures frequently cluster in urban settings and may share overlapping biological pathways—such as oxidative stress, neuroinflammation, and vascular dysfunction—that contribute to cognitive decline and neurodegeneration. As described by Finch and Kulminski, the “Alzheimer’s disease exposome” framework emphasizes the need to assess both endogenous and exogenous environmental factors across time and generations to understand gene-environment-time (G × E × T) interactions in Alzheimer’s etiology [41]. The exposome approach supports more precise modeling of cumulative exposures and life course risks, which is essential for uncovering modifiable environmental contributors to Alzheimer’s disease and related dementias. Furthermore, the recent umbrella review by Jones et al. reinforces this perspective, identifying chronic noise as one of several environmental exposures—alongside fine particulate matter and shift work—linked to increased dementia risk [31]. By enabling simultaneous consideration of multiple exposures, exposomics offers a more holistic and policy-relevant strategy for identifying environmental drivers of dementia.

The associations observed in the above-mentioned studies could also be influenced by other factors. For instance, socioeconomic status (SES) is widely acknowledged in the literature as it can influence both the level of noise exposure and health outcomes [33, 42]. Individuals of lower socioeconomic groups often live in noisier environments and have reduced healthcare access, which may increase their risk of incident ADRD. Notably, only one study from our review did not adjust for SES [28], potentially influencing the accuracy of its reported associations between noise exposure and ADRD.

Further, numerous studies have suggested a role of air pollution in the onset and progression of ADRD [28, 43]; however, it remains less consistently accounted for across studies. Tzivian et al. concluded from their study that air pollution and traffic noise may synergistically influence cognitive function in an adult population [44]. The proposed mechanism suggests that long-term exposure to air pollution can lead to inflammation and oxidative stress, which are implicated in the development of AD [45, 46]. Across numerous studies, particular matter (PM_2.5_) and nitrous dioxide (NO_2_) have most often been linked to cognitive decline and an increased risk of dementia [43, 45, 47, 48]. For instance, Shi et al. reported that prolonged exposure to PM_2.5_ was notably linked with increased incidences of dementia and AD [48]. However, while Carey et al. similarly demonstrate significant associations of air pollutants, such as PM_2.5_ and NO_2_, and night-time noise (L_night_) with incident dementia, a combined model with both air and noise pollutant measurements demonstrated diminished association levels and borderline statistical significance [28]. While these studies suggest an association between air pollution and ADRD, the relationship is complex and not fully understood, indicating the need for further research, especially using exposomic approaches. From our review, four studies adjusted for air pollution measures [22, 24, 25, 28], while three examined joint effects [23, 26, 27]. Some studies may have indirectly captured cumulative exposure associations, but explicit modeling of cumulative burden remains limited in ADRD research.

The exposure assessment models in the reviewed studies demonstrate trade-offs between model complexity, data requirements, and geographic applicability. The sophisticated Danish models (Nord2000) offered detailed environmental integration, while simpler U.S. models prioritized scalability. The deterministic models, while potentially more accurate, require extensive data, which may not always be available. Key differences arose in the level of detail in input data, the sources of noise considered, and the handling of acoustic phenomena. The limitations of each model points to areas for future improvement, including incorporating dynamic traffic data to address temporal constraints; accounting for individual-level factors to reduce exposure misclassification; and developing standardized methodologies like harmonizing key model input variables for cross-study comparisons [49, 50]. By addressing these limitations, future research can improve the accuracy and relevance of noise exposure assessments, facilitating a better understanding of the health impacts of environmental noise.

Additional factors that may influence the association between noise exposure and incident dementia–and should therefore be incorporated in future studies and models–include genetic susceptibility, particularly the presence of the ApoE4 allele, which is a known risk factor for AD. Notably, from our review, Andersson et al. and Weuve et al. are the only studies that adjusted for the ApoE4 allele; however, this adjustment did not alter their baseline findings [22, 27]. Furthermore, other air pollutants beyond NO_2_ and PM_2.5_, such as ozone, should be considered in future models, as they have also been linked to cognitive impairment [51]. Accounting for these variables could improve the accuracy and specificity of models assessing environmental contributions to dementia risk.

This study has several strengths. We used a multilevel modeling framework that accounted for the data’s hierarchical structure and within-study dependencies. We also systematically evaluated effect modification by noise source and dementia subtype and conducted multiple sensitivity and bias assessments to ensure the robustness of findings.

Several limitations must be acknowledged. First, the number of contributing studies was limited, particularly within subgroups, which may reduce power to detect differential effects or publication bias. Second, heterogeneity in noise measurement methods, exposure thresholds, and dementia ascertainment across studies may have introduced variability that was not fully accounted for by model covariates. Third, residual confounding by socioeconomic status, air pollution, or comorbidities cannot be ruled out due to the lack of uniform adjustment in the included studies.

### Conclusion

This meta-analysis found a modest but significant association between long-term noise exposure and an increased risk of ADRD. However, given the limited amount of literature in this field of research, as highlighted by our systematic literature review, the findings of this meta-analysis require constructive interpretation and underscores the need for further research to investigate causal mechanisms and to disentangle effects across different noise sources and ADRD subtypes using standardized exposure assessment methods and large-scale longitudinal data.
