# Supplementary material for: A Comprehensive Systematic Review and Meta-Analysis to Unravel the Noise-Dementia Nexus
Source: Public Health Rev. 2025 Jun 16;46:1607355. doi: 10.3389/phrs.2025.1607355 (PMC12206670; doi:10.3389/phrs.2025.1607355)
Supplement: Supplementary file 1 [file DataSheet1.docx]

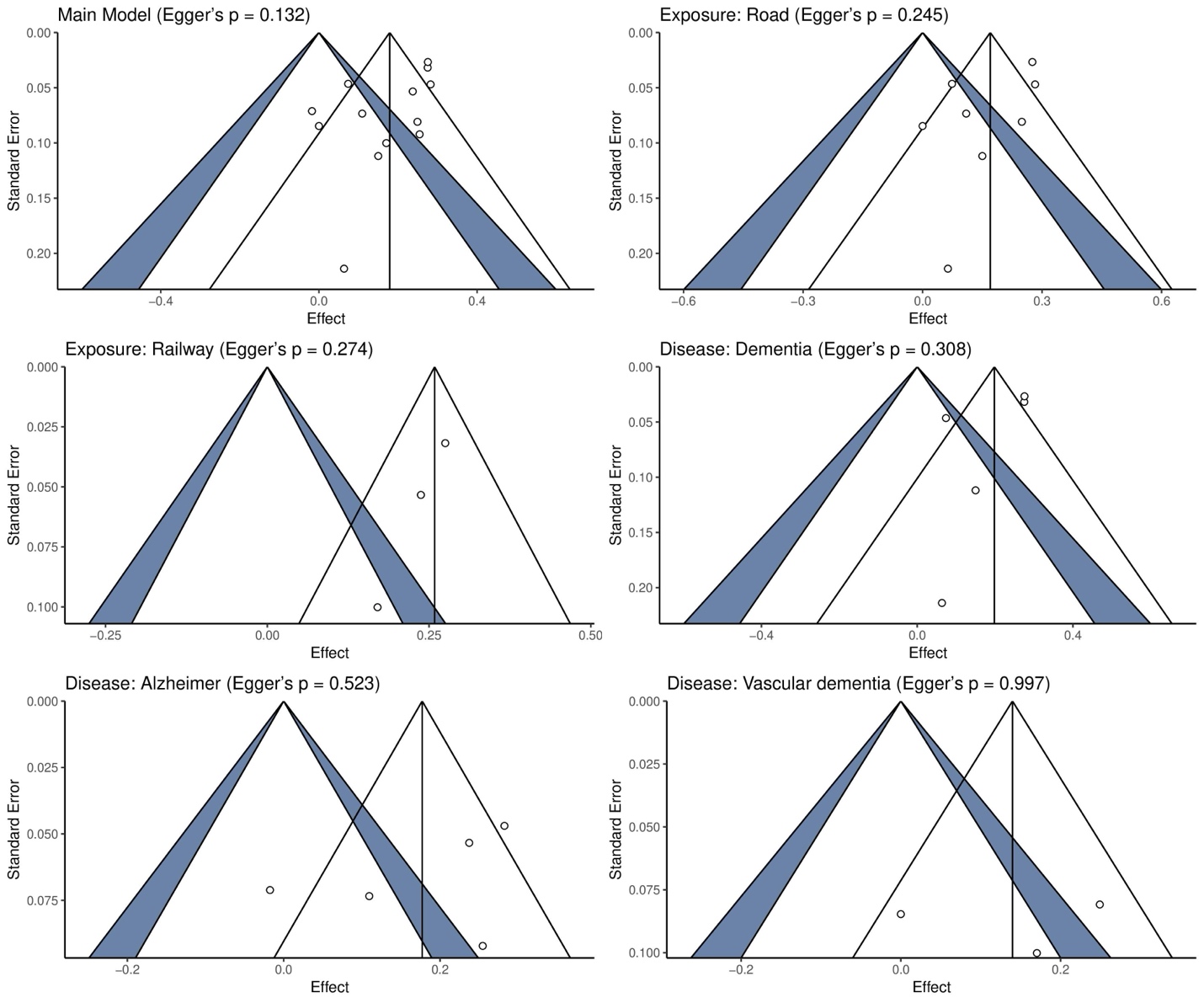


**Supplementary Figure S1. Funnel plots with Egger’s test for publication bias (New York, United States. 2025).**

**Supplementary Table S1. Search strategy and keywords for the current meta-analysis (New York, United States. 2025).**

| **Web of Science**  (TI=(( alzheimer* OR ad OR adrd OR dement* OR "cognitive dysfunction" OR "cognitive decline" OR "cognitive impairment" OR "neurocognitive disorder" OR "mental deterioration" OR "cognitive disorder" OR "Lewy body" OR "frontotemporal dementia" OR "vascular dementia" ) AND ( ( noise OR sound OR "noise pollution" OR "sound pollution" road OR airplane OR airport OR aircraft OR railway OR jet) ))) OR AB=(( alzheimer* OR ad OR adrd OR dement* OR "cognitive dysfunction" OR "cognitive decline" OR "cognitive impairment" OR "neurocognitive disorder" OR "mental deterioration" OR "cognitive disorder" OR "Lewy body" OR "frontotemporal dementia" OR "vascular dementia" ) AND ( ( noise OR sound OR "noise pollution" OR "sound pollution" OR road OR airplane OR airport OR aircraft OR railway OR jet) )) and English (Languages) and Proceeding Paper or Early Access or Book Chapters or Data Paper or Retracted Publication (Exclude – Document Types) and Review Article or  Editorial Material or Meeting Abstract or Book Review or Letter or Note or Correction or Meeting or News Item (Exclude – Document Types) |
| --- |
| **PubMed**  ("Alzheimer Disease"[MeSH Terms] OR "alzheimer*"[Title/Abstract] OR "dement*"[Title/Abstract] OR "Dementia"[MeSH Terms] OR "dementia, vascular"[MeSH Terms] OR "Cognitive Dysfunction"[MeSH Terms] OR "cognitive impairment"[Title/Abstract] OR "cognitive decline"[Title/Abstract] OR "Cognitive Dysfunction"[Title/Abstract] OR "ad"[Title/Abstract] OR “lewy body*”[Title/Abstract] OR "adrd"[Title/Abstract]) AND ("Noise"[Title/Abstract] OR ("Noise"[Title/Abstract] AND "pollution"[Title/Abstract]) OR ("noise, transportation"[MeSH Terms] OR "Noise"[MeSH Terms] OR "noise/adverse effects"[MeSH Terms]) OR "road"[Title/Abstract] OR "airplane"[Title/Abstract] OR "airport"[Title/Abstract] OR  "aircraft"[Title/Abstract] OR "railway"[Title/Abstract] OR "jet"[Title/Abstract] OR "sound"[Title/Abstract]) |
| **Scopus**  ( TITLE ( ( alzheimer* OR ad OR adrd OR dement* OR "cognitive dysfunction" OR "cognitive decline" OR "cognitive impairment" OR "neurocognitive disorder" OR "mental deterioration" OR "cognitive disorder" OR "Lewy body" OR "frontotemporal dementia" OR "vascular dementia" ) AND ( ( noise OR sound OR "noise pollution" OR "sound pollution" OR road OR airplane OR airport OR aircraft OR railway OR jet) ) ) OR ABS ( ( alzheimer* OR ad OR adrd OR dement* OR "cognitive dysfunction" OR "cognitive decline" OR "cognitive impairment" OR "neurocognitive disorder" OR "mental deterioration" OR "cognitive disorder" OR "Lewy body" OR "frontotemporal dementia" OR "vascular dementia" ) AND ( ( noise OR sound OR "noise  pollution" OR "sound pollution" OR road OR airplane OR airport OR aircraft OR railway OR jet) ) ) ) AND ( LIMIT-TO ( DOCTYPE , "ar" ) ) AND ( LIMIT-TO ( LANGUAGE , "English" ) ) |

**Supplementary Table S2. List of excluded studies (New York, United States. 2025).**

| **Authors** | **Year** | **Title** | **Reason for exclusion** | **Details** |
| --- | --- | --- | --- | --- |
| Patel et al. | 2022 | Noise exposure in early adulthood causes age-dependent and brain region-specific impairments in cognitive function | Wrong population | Animal study – excluded based on criteria requiring adult (18+) human populations |
| Dupuis et al. | 2016 | Noise Affects Performance on the Montreal Cognitive Assessment | Wrong exposure | Acute exposure (<1 year); study assessed noise only during testing, failing our criteria for long-term exposure |
| Tzivian et al. | 2020 | The role of depressive symptoms within the association of long-term exposure to indoor and outdoor traffic noise and cognitive function - Results from the Heinz Nixdorf Recall study | Wrong outcomes | Focused on MCI and subtypes, excluding ADRD outcomes |
| Tyas et al. | 2001 | Risk factors for Alzheimer's disease: a population-based, longitudinal study in Manitoba, Canada | Wrong exposure | Self-reported occupational noise exposure; no chronic residential data to assess incidence |
| Ogurtsova et al. | 2023 | Association of long-term air pollution and ambient noise with cognitive decline in the Heinz Nixdorf Recall study | Wrong outcomes | Examined cognitive decline rather than ADRD |
| Stansfeld et al. | 2010 | Night time aircraft noise exposure and children's cognitive performance | Wrong population | Participants aged 9-11 years |
| MacDomhnaill et al. | 2021 | Road traffic noise and cognitive function in older adults: a cross-sectional investigation of The Irish Longitudinal Study on Ageing | Wrong outcomes | Cognitive function tests (i.e. MMSE, MoCA); did not assess ADRD incidence nor prevalence |
| Alimohammadi et al. | 2019 | Relationship Between Noise Annoyance and Cognitive Performance in Automotive Workers Exposed to Chronic Noise | Wrong exposure, wrong outcomes | Occupational noise exposure; evaluated cognitive performance across tests, not ADRD incidence |
| Linares et al. | 2017 | Short-term association between environmental factors and hospital admissions due to dementia in Madrid | Wrong study design | Ecological study design |
| Tzivian et al. | 2016 | Long-Term Air Pollution and Traffic Noise Exposures and Mild Cognitive Impairment in Older Adults: A Cross-Sectional Analysis of the Heinz Nixdorf Recall Study | Wrong outcomes | Focused on MCI rather than ADRD |
| Shehabi et al. | 2022 | The Effect of Lifetime Noise Exposure and Aging on Speech-Perception-in-Noise Ability and Self-Reported Hearing Symptoms: An Online Study | Wrong outcomes | Evaluated hearing outcomes, not ADRD |
| Fuks et al. | 2019 | Road Traffic Noise at the Residence, Annoyance, and Cognitive Function in Elderly Women | Wrong outcomes | Assessed general cognitive function, not ADRD |
| Ghassemzadeh et al. | 2019 | Occupational risk factors for dementia in a sample of older adults covered by the Iranian Oil Industries’ Health Centers, 2018 | Wrong exposure | Occupational exposure; methodology lacks clarity on noise measurement |
| Crous-Bou et al. | 2020 | Impact of urban environmental exposures on cognitive performance and brain structure of healthy individuals at risk for Alzheimer's dementia | Wrong outcomes | Examined cognitive performance and MRI imaging, not ADRD outcomes |
| Chen et al. | 2017 | Living near major roads and the incidence of dementia, Parkinson's disease, and multiple sclerosis: a population-based cohort study | Wrong exposure | Assessed proximity to major roads; did not quantify noise exposure |
| Ju et al. | 2021 | Perceived environmental pollution and subjective cognitive decline (SCD) or SCD-related functional difficulties among the general population | Wrong exposure | Noise was measured qualitatively (“Good” or “Bad”) with self-reported outcomes; not incidence-based |
| Culqui et al. | 2017 | Association between environmental factors and emergency hospital admissions due to Alzheimer's disease in Madrid | Wrong outcomes | Emergency hospital admissions for Alzheimer’s disease, not ADRD incidence |
| Falcón et al. | 2021 | Brain correlates of urban environmental exposures in cognitively unimpaired individuals at increased risk for Alzheimer's disease: A study on Barcelona's population | Wrong outcomes | Whole brain analysis examining gray/white matter volumes, not ADRD incidence |
| Yu et al. | 2020 | Metabolic dysfunction modifies the influence of traffic-related air pollution and noise exposure on late-life dementia and cognitive impairment: A cohort study of older Mexican-Americans | Wrong outcomes | Combined dementia and cognitive impairment outcomes, potentially overestimating ADRD incidence |
| Yu et al. | 2020 | Traffic-related Noise Exposure and Late-life Dementia and Cognitive Impairment in Mexican-Americans | Wrong outcomes | Combined dementia and cognitive impairment outcomes, potentially overestimating ADRD incidence |

**Abbreviations:** MCI, mild cognitive impairment; ADRD, Alzheimer’s disease and related dementias; MMSE, Mini-Mental State Examination; MoCA, Montreal Cognitive Assessment; MRI, magnetic resonance imaging.

**REFERENCES**

Alimohammadi I, Kanrash FA, Abolghasemi J, Vosoughi S, Rahmani K, Chalak MH. Relationship Between Noise Annoyance and Cognitive Performance in Automotive Workers Exposed to Chronic Noise. *J UOEH*. 2019;41(4):375-385. doi:10.7888/juoeh.41.375

Chen H, Kwong JC, Copes R, et al. Living near major roads and the incidence of dementia, Parkinson's disease, and multiple sclerosis: a population-based cohort study. *Lancet*. 2017;389(10070):718-726. doi:10.1016/S0140-6736(16)32399-6

Crous-Bou M, Gascon M, Gispert JD, et al. Impact of urban environmental exposures on cognitive performance and brain structure of healthy individuals at risk for Alzheimer's dementia. *Environ Int*. 2020;138:105546. doi:10.1016/j.envint.2020.105546

Culqui DR, Linares C, Ortiz C, Carmona R, Díaz J. Association between environmental factors and emergency hospital admissions due to Alzheimer's disease in Madrid. *Sci Total Environ*. 2017;592:451-457. doi:10.1016/j.scitotenv.2017.03.089

Dupuis K, Marchuk V, Pichora-Fuller MK. Noise Affects Performance on the Montreal Cognitive Assessment. *Can J Aging*. 2016;35(3):298-307. doi:10.1017/S0714980816000313

Falcón C, Gascon M, Molinuevo JL, et al. Brain correlates of urban environmental exposures in cognitively unimpaired individuals at increased risk for Alzheimer's disease: A study on Barcelona's population. *Alzheimers Dement (Amst)*. 2021;13(1):e12205. Published 2021 Jul 5. doi:10.1002/dad2.12205

Fuks KB, Wigmann C, Altug H, Schikowski T. Road Traffic Noise at the Residence, Annoyance, and Cognitive Function in Elderly Women. *Int J Environ Res Public Health.* 2019;16(10):1790. Published 2019 May 20. doi:10.3390/ijerph16101790

Ghassemzadeh H, Kamrani AA, Momtaz YA, Rassafiani M, Nourhashemi F, Sahaf R, Naderian S. Occupational risk factors for dementia in a sample of older adults covered by the Iranian Oil Industries’ Health Centers, 2018. *Gênero & Direito*. 2019;8(4):482-495. doi:​10.22478/ufpb.2179-7137.2019v8n4.48441

Ju YJ, Lee JE, Lee SY. Perceived environmental pollution and subjective cognitive decline (SCD) or SCD-related functional difficulties among the general population. *Environ Sci Pollut Res Int*. 2021;28(24):31289-31300. doi:10.1007/s11356-021-12831-y

Linares C, Culqui D, Carmona R, Ortiz C, Díaz J. Short-term association between environmental factors and hospital admissions due to dementia in Madrid. *Environ Res*. 2017;152:214-220. doi:10.1016/j.envres.2016.10.020

MacDomhnaill C, Douglas O, Lyons S, Murphy E, Nolan A. Road traffic noise and cognitive function in older adults: a cross-sectional investigation of The Irish Longitudinal Study on Ageing. *BMC Public Health*. 2021;21(1):1814. Published 2021 Oct 8. doi:10.1186/s12889-021-11853-y

Ogurtsova K, Soppa VJ, Weimar C, Jöckel KH, Jokisch M, Hoffmann B. Association of long-term air pollution and ambient noise with cognitive decline in the Heinz Nixdorf Recall study. *Environ Pollut*. 2023;331(Pt 1):121898. doi:10.1016/j.envpol.2023.121898

Patel SV, DeCarlo CM, Book SA, et al. Noise exposure in early adulthood causes age-dependent and brain region-specific impairments in cognitive function. *Front Neurosci*. 2022;16:1001686. Published 2022 Oct 13. doi:10.3389/fnins.2022.1001686

Shehabi AM, Prendergast G, Guest H, Plack CJ. The Effect of Lifetime Noise Exposure and Aging on Speech-Perception-in-Noise Ability and Self-Reported Hearing Symptoms: An Online Study [published correction appears in Front Aging Neurosci. 2023 Aug 28;15:1275798. doi: 10.3389/fnagi.2023.1275798.]. *Front Aging Neurosci*. 2022;14:890010. Published 2022 May 30. doi:10.3389/fnagi.2022.890010

Stansfeld S, Hygge S, Clark C, Alfred T. Night time aircraft noise exposure and children's cognitive performance. *Noise Health*. 2010;12(49):255-262. doi:10.4103/1463-1741.70504

Tyas SL, Manfreda J, Strain LA, Montgomery PR. Risk factors for Alzheimer's disease: a population-based, longitudinal study in Manitoba, Canada. *Int J Epidemiol*. 2001;30(3):590-597. doi:10.1093/ije/30.3.590

Tzivian L, Dlugaj M, Winkler A, et al. Long-Term Air Pollution and Traffic Noise Exposures and Mild Cognitive Impairment in Older Adults: A Cross-Sectional Analysis of the Heinz Nixdorf Recall Study. *Environ Health Perspect*. 2016;124(9):1361-1368. doi:10.1289/ehp.1509824

Tzivian L, Soppa V, Winkler A, et al. The role of depressive symptoms within the association of long-term exposure to indoor and outdoor traffic noise and cognitive function - Results from the Heinz Nixdorf Recall study. *Int J Hyg Environ Health*. 2020;230:113570. doi:10.1016/j.ijheh.2020.113570

Yu Y, Haan M, Paul KC, et al. Metabolic dysfunction modifies the influence of traffic-related air pollution and noise exposure on late-life dementia and cognitive impairment: A cohort study of older Mexican-Americans. *Environ Epidemiol*. 2020;4(6):e122. Published 2020 Dec 3. doi:10.1097/EE9.0000000000000122

Yu Y, Mayeda ER, Paul KC, et al. Traffic-related Noise Exposure and Late-life Dementia and Cognitive Impairment in Mexican-Americans. *Epidemiology.* 2020;31(6):771-778. doi:10.1097/EDE.0000000000001249

**Supplementary Table S3. Multi-level meta-analysis results with and without noise source as a moderator (New York, United States. 2025).**

| **Model** | **Term** | **HR** | **95% CI** | **p-value** | **AIC** | **QE (df)** | **p(QE)** | **QM (df)** | **p(QM)** |
| --- | --- | --- | --- | --- | --- | --- | --- | --- | --- |
| **Baseline (none)** | Intercept | 1.15 | 1.03-1.28 | 0.010 | -22.76 | 40.69 (12) | <0.001 | — | — |
| **Full (noise x disease interaction)** | Intercept (Residential x Alzheimer’s disease) | 1.11 | 0.91-1.36 | 0.29 | 7.67 | 29.08 (6) | <0.001 | 2.38 (6) | 0.881 |
|  | Noise source: Railway | 1.02 | 0.78-1.34 | 0.884 |  |  |  |  |  |
|  | Noise source: Road | 1.07 | 0.83-1.39 | 0.59 |  |  |  |  |  |
|  | Disease: All-cause dementia | 0.98 | 0.90-1.08 | 0.73 |  |  |  |  |  |
|  | Disease: Vascular dementia | 0.94 | 0.81-1.08 | 0.35 |  |  |  |  |  |
|  | Railway x All-cause dementia | 1.05 | 0.91-1.23 | 0.49 |  |  |  |  |  |
|  | Railway x Vascular dementia | 1.00 | 0.77-1.30 | 0.997 |  |  |  |  |  |
| **Additive (noise + disease)** | Intercept (Residential x Alzheimer’s disease) | 1.11 | 0.91-1.36 | 0.29 | -3.2 | 29.49 (8) | <0.001 | 1.79 (4) | 0.773 |
|  | Noise source: Railway | 1.04 | 0.81-1.35 | 0.75 |  |  |  |  |  |
|  | Noise source: Road | 1.06 | 0.82-1.36 | 0.67 |  |  |  |  |  |
|  | Disease: All-cause dementia | 1.00 | 0.93-1.08 | 0.94 |  |  |  |  |  |
|  | Disease: Vascular dementia | 0.94 | 0.83-1.06 | 0.29 |  |  |  |  |  |

**Abbreviations:** *HR*, hazard ratio; *CI*, confidence interval; *AIC*, Akaike information criterion; *QE*, Cochran’s Q statistic for residual heterogeneity; *p(QE)*, p-value associated with QE; *QM*, Q statistic for moderator effects; *p(QM)*, p-value for test of moderators; *df*, degrees of freedom.

**Supplementary Table S4. Leave-one-out sensitivity analysis across meta-regression models (New York, United States. 2025).**

| **Study** | **Model** | **HR** | **95% CI** | **Change in HR** |
| --- | --- | --- | --- | --- |
| Andersson et al, 2018 | Baseline | 1.16 | 1.03-1.30 | +0.01 |
| Cantuaria et al, 2021 | Baseline | 1.1 | 1.0-1.2 | -0.05 |
| Carey et al, 2018 | Baseline | 1.18 | 1.03-1.34 | +0.03 |
| Weuve et al, 2021 | Baseline | 1.13 | 1.0-1.28 | -0.02 |
| Yu et al, 2023 | Baseline | 1.15 | 1.01-1.30 | 0.00 |
| Yuchi et al, 2020 | Baseline | 1.20 | 1.07-1.33 | +0.05 |
| Andersson et al, 2018 | Full | 1.11 | 0.90-1.37 | -0.04 |
| Cantuaria et al, 2021 | Full | 1.11 | 0.93-1.33 | -0.04 |
| Carey et al, 2018 | Full | 1.11 | 0.91-1.36 | -0.04 |
| Weuve et al, 2021 | Full | 0.98 | 0.77-1.26 | -0.17 |
| Yu et al, 2023 | Full | 1.12 | 0.89-1.40 | -0.03 |
| Yuchi et al, 2020 | Full | 1.29 | 0.98-1.69 | +0.14 |
| Andersson et al, 2018 | Additive | 1.11 | 0.91-1.37 | -0.04 |
| Cantuaria et al, 2021 | Additive | 1.11 | 0.93-1.33 | -0.04 |
| Carey et al, 2018 | Additive | 1.11 | 0.91-1.36 | -0.04 |
| Weuve et al, 2021 | Additive | 0.98 | 0.77-1.26 | -0.17 |
| Yu et al, 2023 | Additive | 1.12 | 0.89-1.39 | -0.03 |
| Yuchi et al, 2020 | Additive | 1.29 | 0.98-1.69 | +0.14 |

**Abbreviations:** *HR*, hazard ratio; *CI*, confidence interval.

**Supplementary Table S5. Results of risk of bias assessment with the Newcastle-Ottawa Scale (NOS) for included cohort studies (New York, United States. 2025).**

| **Author, Year** | **NOS** | **Selection** | | | | **Comparability** | **Exposure** | | |
| --- | --- | --- | --- | --- | --- | --- | --- | --- | --- |
|  |  | **Representativeness of exposed cohort** | **Selection of non-exposed cohort** | **Ascertainment of exposure** | **Demonstration that outcome of interest not present at start of study** | **Comparability of cohorts on basis of design or analysis** | **Assessment of outcome** | **Follow-up long enough for outcomes to occur** | **Adequacy of follow-up of cohorts** |
| Cantuaria et al., 2021 | 9 | * | * | * | * | ** | * | * | * |
| Carey et al., 2018 | 8 | * | * | * | * | * | * | * | * |
| Andersson et al., 2018 | 9 | * | * | * | * | ** | * | * | * |
| Weuve et al., 2021 | 9 | * | * | * | * | ** | * | * | * |
| Yu et al., 2023 | 8 | * | * | * | * | * | * | * | * |
| Cole-Hunter et al., 2022 | 8 |  | * | * | * | ** | * | * | * |

**Supplementary Table S6. Results of risk of bias assessment with the Newcastle-Ottawa Scale (NOS) for included case-control studies (New York, United States. 2025).**

| **Author, Year** | **NOS** | **Selection** | | | | **Comparability** | **Exposure** | | |
| --- | --- | --- | --- | --- | --- | --- | --- | --- | --- |
|  |  | **Adequate case definition** | **Representativeness of cases** | **Selection of controls** | **Definition of controls** | **Comparability of cases and controls on basis of design or analysis** | **Ascertainment of exposure** | **Same method of ascertainment for cases and controls** | **Non-response rate** |
| Yuchi et al., 2020 | 8 | * | * | * | * | * | * | * | * |
